# The redox-dependent regulation of satellite cells following aseptic muscle trauma (SpEED): study protocol for a randomized controlled trial

**DOI:** 10.1186/s13063-019-3557-3

**Published:** 2019-07-31

**Authors:** Konstantinos Papanikolaou, Dimitrios Draganidis, Athanasios Chatzinikolaou, Vassiliki C. Laschou, Kalliopi Georgakouli, Panagiotis Tsimeas, Alexios Batrakoulis, Chariklia K. Deli, Athanasios Z. Jamurtas, Ioannis G. Fatouros

**Affiliations:** 1 School of Physical Education, Sport Sciences and Dietetics, University of Thessaly, Karies, 42100 Trikala, Greece; 2 School of Physical Education and Sport Sciences, Democritus University of Thrace, 69100 Komotini, Greece

**Keywords:** Muscle stem cells, Redox potential, Antioxidants, Cell signaling, Tissue regeneration

## Abstract

**Background:**

Muscle satellite cells (SCs) are crucial for muscle regeneration following muscle trauma. Acute skeletal muscle damage results in inflammation and the production of reactive oxygen species (ROS) which may be implicated in SCs activation. Protection of these cells from oxidative damage is essential to ensure sufficient muscle regeneration. The aim of this study is to determine whether SCs activity under conditions of aseptic skeletal muscle trauma induced by exercise is redox-dependent.

**Methods/design:**

Based on the SCs content in their vastus lateralis skeletal muscle, participants will be classified as either high or low respondents. In a randomized, double-blind, crossover, repeated-measures design, participants will then receive either placebo or *N*-acetylcysteine (alters redox potential in muscle) during a preliminary 7-day loading phase, and for eight consecutive days following a single bout of intense muscle-damaging exercise. In both trials, blood samples and muscle biopsies will be collected, and muscle performance and soreness will be measured at baseline, pre-exercise, 2 and 8 days post exercise. Biological samples will be analyzed for redox status and SCs activity. Between trials, a 4-week washout period will be implemented.

**Discussion:**

This study is designed to investigate the impact of redox status on SCs mobilization and thus skeletal muscle potential for regeneration under conditions of aseptic inflammation induced by exercise. Findings of this trial should provide insight into (1) molecular pathways involved in SCs recruitment and muscle healing under conditions of aseptic skeletal muscle trauma present in numerous catabolic conditions and (2) whether skeletal muscle’s potential for regeneration depends on its basal SCs content.

**Trial registration:**

ClinicalTrials.gov, ID: NCT03711838. Registered on 19 Oct 2018.

**Electronic supplementary material:**

The online version of this article (10.1186/s13063-019-3557-3) contains supplementary material, which is available to authorized users.

## Background

Skeletal muscle stem cells (satellite cells – SCs) are required for muscle repair, remodeling and growth [1]. Under conditions of increased transcriptional activity, such as in response to skeletal muscle injury, SCs are activated to promote healing [2]. SCs can be identified by their location (beneath the basal lamina of myofibers) and by the expression of transcriptional proteins, such as paired box protein (Pax7). Each stage of the regeneration process is partly controlled by myogenic regulatory factors (MRFs) which include myogenic factor 5 (Myf5), myogenic determination factor (MyoD), myogenin and myogenic factor 4 (Myf4), all orchestrating skeletal muscle’s myogenic programming [3, 4].

Recent data suggest a redox-regulated link between SCs and myogenesis [5]. Muscle injury triggers the nuclear factor kappa-light-chain-enhancer of activated B cells (NF-kB)-mediated apoptosis to control stem-cell survival and maintain stemness [6]. SCs’ antioxidant reserves protect these cells from oxidative damage whereas antioxidant enzymes, glutathione peroxidase 3 (GPX-3), superoxide dismutase 2 (SOD-2) and thioredoxin (Trx-1) regulate the homeostatic programming of quiescent SCs [7, 8]. Redox-sensitive signaling pathways, such as Notch and Wnt/β-catenin, are also critical for multiple stages of myogenesis including activation and proliferation of SCs. Interestingly, several Wnt proteins have been shown to regulate SCs activation, proliferation and differentiation [9, 10].

Skeletal muscle trauma is observed in numerous catabolic conditions, characterized by elevated proteolysis and muscle wasting, such as cancer, cachexia and muscular dystrophy, which result in physical capacity impairment and a deteriorated quality of life [11, 12]. In the absence of SCs, injured skeletal muscle abolishes its ability to regenerate or regenerates very poorly in response to muscle injury [13]. Flow cytometric analysis in isolated SCs revealed that the number of Pax7^+^ cells/mg of muscle in G2/M, G0/G1 and S phases of the cell cycle increased by 202%, 32% and 59%, respectively, at 24 h post injury [14]. Under these conditions, SCs rapidly migrate to the injured area, differentiate into mature myoblasts and contribute to myofiber repair [15]. Exercise-induced muscle damage (EIMD) results in an aseptic muscle microtrauma and a pronounced inflammatory cascade characterized by leukocyte immobilization, macrophage infiltration, cytokine production and increased oxidative stress (OXS) [16, 17]. A study incorporating a muscle damage protocol (300 maximal eccentric contractions) on an isokinetic dynamometer, showed an increase in SCs content even at 180 h post exercise [18]. These marked similarities between aseptic EIMD and trauma makes eccentric exercise a valuable model to investigate the redox-dependent intracellular regulation of SCs involvement in skeletal muscle’s healing potential.

Only two studies have examined the effectiveness of antioxidant supplementation on skeletal muscle healing and the inflammatory response in primary cultured cells and rat gastrocnemius muscle demonstrating that polyphenol administration enhances SCs activation and evokes an earlier appearance of M2 macrophages promoting an anti-inflammatory phenotype [19, 20]. However, no studies have investigated, so far, the effect of redox status perturbations on SCs responses and myogenic potential following aseptic trauma in human skeletal muscle. A powerful antioxidant that has been in clinical and sports performance practice for several decades is *N*-acetylcysteine (NAC). NAC is a thiol donor, non-specific antioxidant with a binary antioxidant role: Firstly, NAC directly scavenges a number of reactive oxygen species (ROS) and secondly, it produces reduced cysteine (Cys) by deacetylation, which supports the biosynthesis of endogenous reduced glutathione (GSH) via the activity of γ-glutamylcysteine synthase [21]. GSH plays a pivotal role in cellular metabolism and especially in terms of stem cells’ oxidative defense, activity, survival and self-renewal under conditions of increased OXS and ROS production [22]. Low to moderate ROS levels control physiological cellular signaling pathways and facilitate muscle growth and development while high ROS levels impair myogenesis and cause cell death through apoptotic and/or necrotic mechanisms (e.g., NF-kB signaling) [23]. We hypothesize that NAC administration following aseptic muscle trauma, characterized by severe inflammation and high levels of ROS, will alleviate the increase in oxidative stress by decreasing ROS activity levels, resulting in an optimal intracellular redox environment, favoring the activity (activation, proliferation, differentiation and fusion) of SCs and thus facilitating muscle’s regenerative process and adaptation following muscle injury. Thus, the objectives of this clinical trial are to examine the effects of redox status perturbation (via NAC administration) on the intracellular pathways responsible for SCs responses following aseptic muscle trauma induced by damaging exercise.

## Methods/design

### Study overview and design

The methods and ethics of the present study have been approved by the Institutional Review Board of the University of Thessaly (ref. number 1387) and procedures are in accordance with the Declaration of Helsinki, as revised in 2013. The SpEED study incorporates a randomized, two-trial (NAC vs placebo), cross-over, double-blind, repeated-measures design. Participants who will fulfill the inclusion criteria will have their body mass, height, resting metabolic rate (RMR), body composition, muscle strength, soreness and maximal oxygen consumption (VO_2max_) measured. Since protein and antioxidant consumption as well as systematic physical activity (PA) and/or exercise may affect SCs responses and/or redox status, participants’ daily nutrient intake (via a 7-day diet recall with emphasis on protein and antioxidant intake) and PA levels (via accelerometry instrumentation) will be monitored for a week before the inception of the study [24–26]. Then, a 2-week adaptation period (only before the first trial) will be applied to adjust (through a balanced diet, using RMR values and daily energy expenditure measurements) protein and antioxidant intake at the levels required by current Recommended Dietary Allowances (RDAs) (0.8–1 g of protein/kg/day; 900 mg/day of vitamin A; 90 mg/day of vitamin C; 600 IU/day of vitamin D; 15 mg/day of vitamin E; 11 mg/day of zinc; 400 mg/day of magnesium and 55 mg/day of selenium) [27, 28]. This dietary protocol will be designed and implemented by a registered dietician. During the same time-frame, familiarization with experimental procedures will be provided.

Evidence exists that skeletal muscle growth and myogenic potential is directly related to its basal SCs content [24, 29]. As such, volunteers will provide a resting (they will abstain from any exercise or intense PA for at least 5 days prior to sampling) muscle biopsy sample (baseline sampling) from their vastus lateralis muscle (of their dominant limb) and based on its SCs content will be stratified to either high (HR) or low (LR) respondents. Participants will be stratified as HR or LR using a K-means cluster analysis. Blood sampling and performance measurements will also be performed at baseline.

**Fig. 1:**
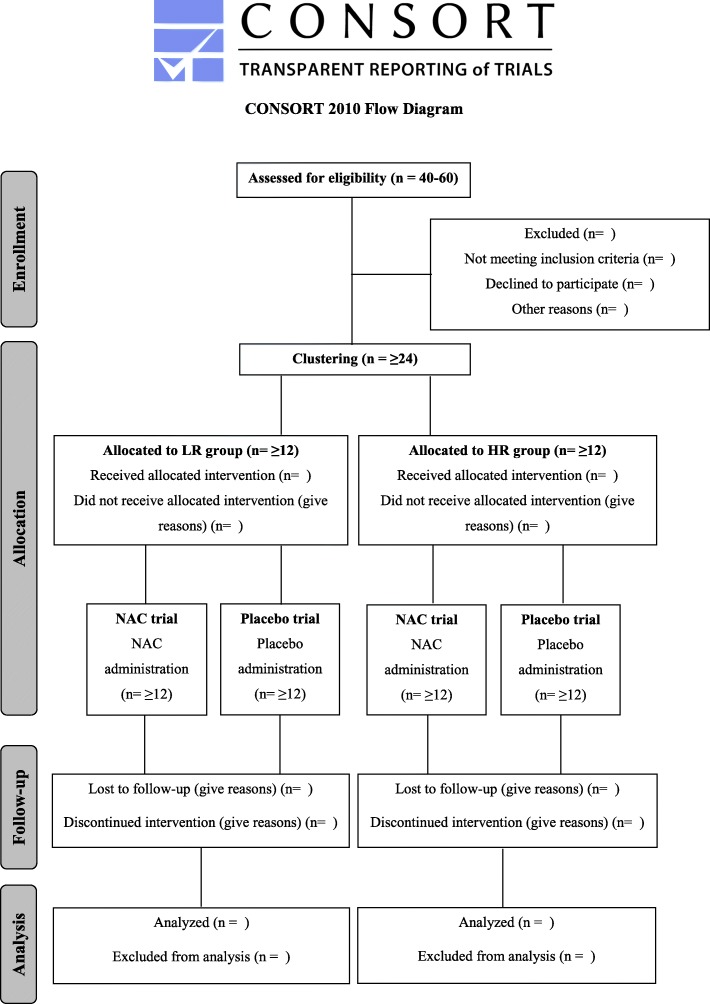
The Consolidated Standards of Reporting Trials (CONSORT) diagram of the study

**Fig. 2:**
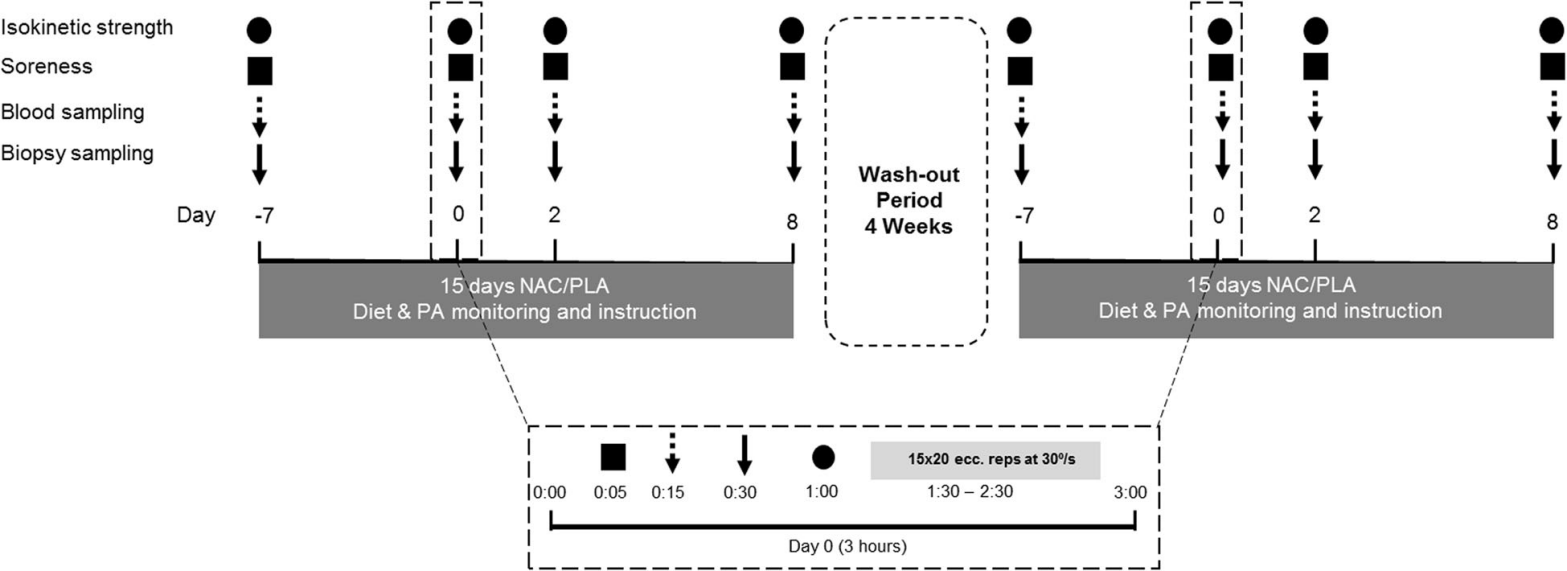
The experimental flowchart for the clinical trials

Following stratification to the HR and LR groups, volunteers of each group will then perform two trials in a randomized order: (1) NAC ingestion and (2) placebo ingestion. NAC or placebo will be consumed before (a 7-day loading phase) and after (immediately post exercise and for eight consecutive days thereafter) an intense eccentric exercise protocol. During both trials, participants will follow the balanced daily dietary protocol of the adaptation period. However, daily dietary intake will be recorded and analyzed during each trial in an attempt to minimize deviations from the prescribed diet. A 4-week washout period will be implemented between trials (dietary intake during this period will also be adjusted according to that applied during the adaptive period). During the entire experimental period, participants will be asked to abstain from any strenuous PA or exercise. Muscle biopsies and blood samples will be collected after overnight fasting before the exercise protocol (pre-exercise sampling) as well as at 2 and 8 days post exercise. Muscle strength and delayed onset of muscle soreness (DOMS) will be evaluated at the same time points. All measurements and collection of biological samples will be performed at the same time of day, in both trials, to prevent circadian rhythm variations. Figure 1 shows the Consolidated Standards of Reporting Trials (CONSORT) diagram of the study and Fig. 2 illustrates the experimental flowchart for the clinical trials.

The primary outcomes of the study are the SCs-specific markers measured in muscle (Pax7^+^, MyoD^+^ cells per type I/II myofibers), the macrophages’ markers measured in muscle (cluster of differentiation 11b marker (CD11b^+^), cluster of differentiation 206 marker (CD206^+^) cells per myofiber), the myogenesis-related markers measured in muscle (Myf5, MyoD, myogenin, MRF4 and myostatin mRNA expression levels), the cell signaling markers measured in muscle (GPX-3, SOD-2, Trx-1, insulin-like growth factor 1 (IGF-1), Notch1, Wnt3 protein expression levels), and the oxidative stress markers measured in muscle (GSH, oxidized glutathione (GSSG), protein carbonyls (PC) and malondialdehyde (MDA)). The secondary outcomes are the oxidative stress markers measured in blood (total antioxidant capacity (TAC) in serum, GSH, GSSG, catalase (CAT) and hemoglobin (Hb) in red blood cell lysates), and the inflammatory markers measured in blood (C-reactive protein (CRP), tumor necrosis factor alpha (TNF-α), interleukin (IL)-6, IL-8, IL-10), blood creatine kinase activity (CK) as a marker of muscle damage and serum cortisol). Other outcomes include body mass, height, body mass index (BMI), total and regional fat mass, lean mass and body fat, RMR, number of steps/day and time spent at sedentary, light, moderate, vigorous and moderate-to-vigorous PA, total daily energy intake and expenditure, daily intake of carbohydrate, fat, protein, vitamin A, vitamin C, vitamin D, vitamin E, selenium, zinc and magnesium, VO_2max_, knee extensors’ (KE) maximal eccentric and concentric peak torque and DOMS.

### Participants recruitment and screening

We will initially recruit 40–60 young men. In organized meetings, participants will be informed by the investigators about the purpose of the study, the experimental procedures and all the possible risks and benefits associated. Participants will be recruited via media advertisements and posters. All volunteers will complete a health history questionnaire and a written consent form will be acquired from each participant by the investigators. All personal information and data obtained will be confidential and only the researchers of the study will have access to the database. Participants will be included in the study if they (1) are healthy, non-smokers, aged 18–30 years; (2) have a BMI of 18.5–24.9 kg/m^2^; (3) abstain from any vigorous PA during and ≥ 4 weeks prior to the study; (4) have no recent history of musculoskeletal injury, lower limb trauma and metabolic diseases; and (5) they refrain from consumption of alcohol, caffeine, any type of nutritional supplements, non-steroidal anti-inflammatory drugs (NSAIDs) and medication before (≥ 6 months) and throughout the experimental period.

### Exclusion criteria

- Allergies or intolerance to NAC
- Recent febrile illness
- Use of anti-inflammatory medication
- Use of medication interacting with muscle metabolism

### Exercise protocol

Participants will perform a protocol consisting of 300 eccentric unilateral maximal contractions (20 sets, 15 repetitions/set, 30-s rest between sets) of the quadriceps muscles on an isokinetic dynamometer (Cybex 770, USA) at a speed of 30°/s. A different limb will be used in each trial. Before the protocol a standard warm up will precede involving 8-min cycling on a cycle ergometer (Monark 834, 154 ERGOMED C, Sundbyberg, Sweden) at a speed of 70 rpm/min and at 50 W, followed by 5-min stretching exercises. KE will be isolated using straps in the shoulders, hips and thigh. This protocol has been described in the literature to effectively induce a significant level of skeletal muscle damage and myofibrillar disruption as documented with electron microscopy and immunohistochemistry [30, 31].

### Supplementation protocol

Participants will consume either NAC or placebo in a random order according to a double-blind, crossover design. A dosage of 40 mg NAC/kg/day will be administered orally in three doses (equally distributed), in order to maximize cysteine levels (for glutathione synthesis) in the circulation and skeletal muscle, primarily due to NAC fast rate of clearance (~ 6 h post ingestion) [32]. According to a model proposed by Reid in 2001, there is an optimal intracellular redox status required for optimum muscle function and force production [33]. Hence, when NAC is supplemented orally as an exogenous antioxidant (glutathione precursor), under conditions of increased oxidative stress and ROS production, it is plausible to administer a moderate dose for optimal scavenging of ROS, which according to recent studies, represents an absolute dose of ~ 1.2 to 5 g/day and evokes a pronounced improvement in muscle function and performance [34–37]. Larger doses of NAC supplementation may buffer physiological levels of cellular oxidants and may result in impaired muscle force production and performance deterioration [38]. With our dose of 40 mg/kg/day we will reach an absolute dose of approximately ~ 2.8 g of NAC for an average participant weighted ~ 70 kg. Additionally, this dosage has been shown to successfully increase total thiol levels in plasma [39]. NAC in a powder form will be diluted in a 250-ml drink containing 248 ml water and 2 ml of natural, non-caloric, flavoring-sweetener containing sucralose (Flavdrops, My Protein, Cheshire, UK). The placebo supplement will be prepared to be identical to NAC in terms of taste and smell apart from the NAC content. In both trials, each participant will be asked 15 times (once a day) if they realize whether the drink they consumed was the placebo or the experimental one. Responses will be recorded, and correct or incorrect answers will be measured. A research assistant will perform the randomization and assign participants to the interventions (NAC vs placebo) during the clinical trials using an online, computer-based, third-party, semi-automatic randomization system. The same research assistant will have full access to this list and will monitor the presence of any adverse side effects in both trials via questionnaires [30]. Both investigators and participants will be blinded to supplementation condition. Possible adverse reactions to oral NAC supplementation include an upset stomach, nausea, stomach and/or intestinal gas, sleepiness, metallic taste, light-headedness, redness of eye, face, or hands, and cough [39].

### Anthropometric measurements

Standing body mass and height will be measured on a beam balance equipped with stadiometer (Beam Balance-Stadiometer, SECA, Vogel & Halke, Hamburg, Germany) while participants wear light clothing as described previously [40]. BMI will be calculated as mass per height squared. Dual energy X-ray absorptiometry (DXA) scanner (GE Healthcare, Lunar DPX-NT, Singapore) will be utilized for body composition assessment. On each testing day the equipment will be calibrated using a LS phantom in accordance to standard procedures. Participants will be asked to remain still and they will be scanned in the supine position using the total body analysis under scanning conditions automatically selected by the software (standard, thick, thin scanning). Total and regional fat mass (g), lean mass (kg) and body fat (% and kg) values will be obtained. GE enCORE software will be utilized for all DXA scans and analyses.

### Resting metabolic rate

For RMR assessment, resting VO_2_/VCO_2_ values will be measured in the morning (07:00–09:00) after overnight fasting utilizing an open-circuit type indirect calorimeter with a ventilated hood system (Vmax Encore 29, BEBJO296, Yorba Linda, CA, USA) and the 24-h RMR will be calculated as previously described [41].

### Physical activity assessment

Habitual PA will be monitored over a 7-day period using the ActiGraph, GT3X+ accelerometers (ActiGraph, Pensacola, FL, USA). Participants will be taught, by an experienced researcher, how to wear the adjustable belt on the waist with the accelerometer monitor on the right side of the hip and they will be asked to wear it throughout the day for seven consecutive days, apart from bathing, swimming and sleeping. To be included in the analysis, participants will have to complete four full days of wearing time (i.e., ≥ 4 days with ≥ 10 wear hours/day). From the data obtained, non-wear time will be calculated and daily activity levels and sedentary time will be expressed as steps per day and time spent at sedentary, light, moderate, vigorous and moderate-to-vigorous PA [42, 43]. ActiLife 6 software will be used to initialize accelerometers and download data using a 60-s epoch length.

### Dietary intake analysis

Participants will be instructed by a registered dietitian on how to estimate food/fluid servings and sizes and how to complete a 7-day diet recall before and during both trials and the washout period to ensure that they will follow the same dietary regimen. Specifically, participants will be provided with colored images showing different food portions and detailed instructions that they will use to weight their food. When possible, the name of the brand and/or manufacturer will be recorded. Diet recalls will be analyzed using the Science Fit Diet 200 A (Science Technologies, Athens, Greece) dietary software for data regarding total energy (kcal), carbohydrate, fat, protein (g/kg/day and g/day), vitamin A (mg/day), vitamin C (mg/day), vitamin D (IU/day), vitamin E (mg/day), selenium (mg/day), zinc (mg/day) and magnesium (mg/day).

### Maximal oxygen consumption

VO_2max_ will be measured using open-circuit spirometry with an automated pulmonary gas exchange system (Vmax Encore 29, BEBJO296, Yorba Linda, CA, USA) via the breath-by-breath analysis during a graded exercise test on a treadmill (Stex 8025 T, Daegu, Korea) until volitional fatigue, according to procedures previously described [44]. Briefly, following a standard warm-up (8 min of low-intensity running on a treadmill) each participant will complete a graded exercise test protocol at a starting speed of 8–10 km/h (depending on participants’ fitness training history), with an increase of 1 km/h in the running speed every 2 min. During the test VO_2_/VCO_2_ values will be measured in 20-s intervals. Criteria for terminating the test include: (1) participant reached a level of volitional fatigue, (2) predicted maximum heart rate reached and/or surpassed, (3) respiratory quotient values ≥ 1.10 and (4) plateau in VO_2_ values. VO_2max_ will be calculated from the averaged VO_2_ measures during the final minute of the test.

### Muscle strength and soreness

KE maximal eccentric and concentric peak torque of the exercised limb will be measured on an isokinetic dynamometer (Cybex 770, Rosemont, IL, USA) at 60°/s as described elsewhere [45]. DOMS of KE of the exercised limb will be evaluated by palpation of the belly and distal region after participants have performed three full-squat repetitions. Then, participants will rate their soreness level on a scale from 1 to 10 (1 = no pain, 10 = extremely sore). DOMS assessments will be carried out by the same investigator [46].

### Blood sampling and biochemical assays

Following an overnight fasting blood samples will be drawn from the antecubital vein by venipuncture with a 20-gauge disposable needle equipped with a Vacutainer® tube holder (Becton Dickinson, Franklin Lakes, NJ, USA) with the participants in a supine position. For serum separation, blood samples will be allowed to clot at room temperature and then will be centrifuged (1370 g, 10 min, 4 °C). The supernatant will be aliquoted into eppendorf tubes for subsequent analysis of CRP, TNF-α, IL-6, IL-8, IL-10 (inflammation), CK activity (muscle damage), TAC (oxidative stress) and cortisol (hormonal response). Another blood portion will be collected in ethylenediaminetetraacetic acid (EDTA)-containing tubes and will be centrifuged at 1370 g, 10 min, 4 °C to collect the plasma. Plasma samples will be used for the measurement of PC (protein oxidation) and MDA (lipid peroxidation). Packed erythrocytes (RBCs) will be obtained after lysis of the plasma samples for the measurement of GSH, GSSG, CAT and Hb (RBCs’ redox status). All samples will be aliquoted in multiple eppendorf tubes and stored at − 80 °C until analysis. A small portion of whole blood (~2 ml) will be collected in tubes containing EDTA for a complete blood count analysis on an automated hematology analyzer (Mythic 18, Orphee SA, Geneva, Switzerland). All assays will be performed in duplicate.

### Muscle biopsy sampling

Percutaneous needle muscle biopsies will be obtained after an ~ 10 h overnight fast (baseline) using the Bergstrom technique with the application of manual suction, from the mid-portion of the vastus lateralis muscle under local anesthetic (xylocaine 1%), by a registered surgeon [47]. After the biopsy, no antibiotics, pain killers or anti-inflammatory drugs will be administered to participants. Volunteers who will receive any type of pharmaceutical drugs and/or analgesics in the biopsy site, in the rare case of excess bleeding or pain, will be excluded from the analyses. Subjects will be asked to refrain from any PA at least 96 h prior to muscle biopsy sampling. Subsequent muscle biopsies (pre exercise, 2 and 8 days post exercise) will be spaced 5 cm apart to diminish a repeated biopsy effect. Upon excision, adipose tissue and blood will be carefully removed and muscle samples suited for histology will be aligned and immediately be mounted in optimal cutting temperature (OCT) compound, immersed in nitrogen-cooled isopentane and stored at − 80 °C. Embedded samples will be sectioned (7 μm) at − 20 °C using a cryostat, placed on glass slides and stored at − 80 °C. Muscle samples suited for mRNA, Western blotting, muscle thiols and OXS analyses will directly be frozen in liquid nitrogen, and stored at − 80 °C.

### Histological analyses

Sections will be stained with hematoxylin and eosin (H&E) in order to quantify damaged myofibers [48]. Myofibers indicating loss of the physiological outline, sarcolemmal damage, mononuclear cell infiltration and centrally located nuclei will be expressed as a percentage of the total number of myofibers.

### Immunofluorescence

Muscle cross-sections (7 μm) will be allowed to air dry at room temperature for 30 min. For fiber-type-specific SCs analyses, samples will be stained with appropriate primary and secondary antibodies against specific antigens such as, Pax7, MyoD, myosin heavy chain type II, and laminin as described previously [49–51]. For fiber-type-specific M1 (pro-inflammatory) and M2 (anti-inflammatory) macrophage quantification, muscle cross-sections will be stained with appropriate primary and secondary antibodies against CD11b^+^, CD206^+^ and laminin as described previously [52]. Nuclei will be visualized with 4′,6-diamidino-2-phenylindole (DAPI) contained in the mounting media prior to coverslipping. The specificity of staining will be verified using negative controls. Slides will be viewed using an Olympus BX41 microscope equipped with appropriate filters and a high-resolution fluorescent camera. Images will be captured and analyzed using the Image-Pro Plus v6.0 software. All images will be obtained with the 20X objective. Myofiber cross-sectional area (CSA), fiber type distribution (% type I and II fibers), myonuclei content (DAPI^+^ cells), fiber-type-specific SCs content and activation status (Pax7^+^ and MyoD^+^ cells per myofiber) and M1 (CD11b^+^/CD206^–^cells) and M2 (CD11b^+^/CD206^+^ cells) macrophage content will be determined. The SCs content and activation will be determined via the colocalization of Pax7 and DAPI and/or the colocalization of Pax7, MyoD and DAPI within the laminin border. M1 and M2 macrophage content will be determined via the colocalization of CD11b, CD206 and DAPI.

### Quantitative RT-PCR

Total RNA will be isolated from 10–20 mg of frozen muscle tissue using the NucleoSpin RNA Plus kit (Machery-Nagel, Bethlehem, PA, USA), according to the manufacturer’s instructions, at a final volume of 80–120 μL. RNA concentration (ng/ml) and purity (260/280) will be measured spectophotometrically (Hitachi UV/VIS; Hitachi Instruments Inc., Tokyo, Japan). Then samples will be reverse transcribed using a PrimeScript 1st strand cDNA synthesis kit (Takara Mountain View, CA, USA) in 20-μl reaction volumes, according to the manufacturer’s protocol. Quantitative RT-PCR reactions will run in triplicates containing RT Sybr Green qPCR Master Mix. Primers for Myf5, MyoD, myogenin, MRF4, myostatin and gyceraldehyde 3-phosphate dehydrogenase (GAPDH) will be purchased and mRNA expression levels will be calculated using the 2^−ΔΔCt^ method. Fold changes from baseline will be calculated using the ΔΔC_t_ method and normalization will be performed using the housekeeping gene GAPDH [53].

### Western blotting

Changes in protein expression levels of GPX-3, SOD-2 and thioredoxin Trx-1 (related to SCs homeostasis), IGF-1, Notch1 and Wnt3 (related to SC mobilization) will be analyzed by immunoblotting. Muscle samples will be homogenized in lysis buffer and then centrifuged (13,000 rpm, 4 °C, 10 min) and the supernatant will be collected. Total protein concentration will be determined using the Bradford method (Bradford Protein Assay; Bio-Rad). Twenty milligrams of protein will be loaded in gradient precast gels (Mini-PROTEAN TGX Gels; Bio-Rad, Hercules, CA, USA) and will be subjected to SDS-PAGE electrophoresis at room temperature. Afterwards, proteins will be transferred to trans-blot stacks using the Trans-blot Turbo transfer system (Bio-Rad, Hercules, CA, USA), blocked for 1 h and incubated with primary antibodies overnight at 4 °C. Membranes will be washed in tris-buffer saline (TBS-T) solution and will be incubated with appropriate secondary antibodies for 1 h at room temperature. Following another washing step (in TBS-T), membranes will be visualized by chemiluminescence and quantified using densitometry. Normalization will be performed with the housekeeping protein GAPDH.

### Muscle thiols and OXS markers

Muscle samples will be homogenized in phosphate buffer saline (PBS) containing protein inhibitors as described previously [54]. After homogenization, the samples will be centrifuged (12,000 g, 4 °C, 30 min) and the supernatant will be collected. GSH, GSSG, PC and MDA levels will be measured as indices of muscle’s redox status. All measurements will be performed spectophotometrically (Hitachi UV/VIS; Hitachi Instruments Inc., Tokyo, Japan) as described elsewhere [54]. All assays will be performed in duplicate.

### Statistical analyses and Power calculation

A preliminary power analysis (based on previous studies that used NAC administration to investigate its effects on EIMD), using the G*Power 3.0.10 program, showed that a minimum number of 10 participants per group is needed to obtain statistical meaningful results among repeated measurements [30]. Specifically, power calculation was performed for a two-way repeated-measures analysis of variance (ANOVA), within-between interaction test and input variables included: effect size, 0.55; α error, 0.05; power, 0.95; number of groups (LR and HR), 2; correlation among repeated measures, 0.5 and non-sphericity correction, 1. However, the total number of participants depends also on potential dropouts according to the following formula: *n* ’  = n/(1 − d) [55].

Thus, the final number of participants to be recruited with a dropout rate of 15% would be *n*’ = 10/(1–0.15) = 11.8. Therefore, ≥ 12 participants per group (LR vs HR) will be selected from the initial sample (*N* = 40–60) via k-means clustering to participate in the clinical trial (NAC vs placebo).

A k-means cluster analysis will be utilized to efficiently define two separate groups of subjects (LR and HR groups) from the total sample (*N* = 40–60), based on the SCs content of their vastus lateralis muscle of their dominant leg [24]. This type of analysis requires a relatively large initial sample size (*N* = ≥ 40), is a form of partitional clustering and is a multivariate method used to identify homogeneous groups (i.e., clusters) of cases based on a common trait [29, 56].

All analyses and reporting of the results will comply with the Standard Protocol Items: Recommendations for Interventional Trials (SPIRIT) Statement for reporting randomized clinical trials (RCTs) [57]. Results of participant’s baseline characteristics and outcome variables (primary, secondary and other) will be summarized using descriptive statistics and will be expressed as mean (standard deviation) or median (range) for continuous variables. Data normality will be examined using the Kolmogorov-Smirnov and the Shapiro-Wilk tests. If our data sets follow normal distribution, parametric tests will be applied. Baseline comparisons on the LR and HR groups (anthropometrics, body composition, strength, VO_2max_, dietary profile, PA) will be performed using a one-way ANOVA test. Time- and trial-effect comparisons within and between trial (NAC or placebo) in the LR and HR groups will be analyzed using a two-way repeated-measures ANOVA test with a Bonferroni correction for pairwise comparisons. If the data normality is violated, non-parametric tests will be applied. Baseline comparisons on the LR and HR groups will be performed using a Kruskal-Wallis test. Time-effect comparisons within trial (NAC or placebo) in the LR and HR groups will be analyzed using a Friedman test accompanied by Wilcoxon signed-rank test for pairwise comparisons. Trial-effect comparisons between trials (NAC vs placebo) in the LR and HR groups will be analyzed using a Kruskal-Wallis test accompanied by a Mann-Whitney *U* test for pairwise comparisons. Pearson’s correlation analysis will also be used to examine possible relations among variables. Correlation coefficients of r < 0.2, 0.2 < r < 0.7 and r > 0.7 will be defined as small, moderate and high, respectively. The level of statistical significance will be set at *p* < 0.05. Effect sizes (ES) and confidence intervals (95% CI) will be calculated on results of all dependent variables using the Hedge’s *g* method, corrected for bias. ES will be interpreted as none, small, medium-sized and large for values 0.00–0.19, 0.20–0.49, 0.50–0.79 and ≥ 0.8, respectively. Multiple-imputation analysis will also be utilized to handle missing data during data collection and sensitivity analyses will be executed to evaluate the robustness of the results [58]. Statistical analyses will be performed using the SPSS 20.0 software (IBM Corp., Armonk, NY, USA).

## Discussion

The present study is designed to assess the impact of redox status on SCs responses and the mechanisms (hormonal regulation, M1 and M2 macrophages, intracellular signaling) associated with their mobilization and function following aseptic skeletal muscle trauma induced by exercise. Nutritional supplementation or medications have been shown to affect SCs biology under traumatic conditions. Hydrolyzed whey protein supplementation and anti-inflammatory medication (ibuprofen) results in increased SCs response and expedites skeletal muscle recovery [25, 48]. Antioxidant supplementation (vitamin C, vitamin E, NAC or combined antioxidants) may attenuate loss of muscle force production and reduce muscle soreness and lipid peroxidation levels but it may also delay recovery suggesting a potential redox-associated mechanism involved in muscle healing [54, 59]. However, this possibility has not been explored in the human skeletal muscle. More specifically, there is no information regarding the redox-dependent mechanism of SCs mobilization and action in human skeletal muscle. It is well established in the literature that thiol oxidation is a major post-translational oxidative modification affecting the cysteine residues in multiple proteins [60]. On the other hand, increased ROS can oxidize GSH leading to irreversible modification [61]. In this sense, low levels of GSH may not only attenuate the antioxidant defense leading to SCs damage but it may also alter cellular redox status drastically. Furthermore, SCs activity is also determined by redox-sensitive cues (cytokines, immune cells, signaling molecules) emerging from the surrounding microenvironment [62]. Supplementation with a powerful GSH precursor, such as NAC, could not only foster GSH levels and thus muscle’s antioxidant potential but also will change myofiber redox balance which is crucial for the redox-dependent regulation of intracellular signaling pathways mediating pro- and anti-inflammatory response to muscle trauma [30]. Our research hypothesis states that NAC-induced change of muscle’s redox status will upregulate SCs availability and mobility under conditions of increased oxidative stress and inflammation in human skeletal muscle. This is of great importance as muscle injury is present in several clinical conditions characterized by increased muscle wasting, atrophy and sepsis that result in physical deterioration and poor quality of life such as in many types of cancer, cachexia, muscular dystrophies, etc. [11, 12]. Consumption of NAC, a potent thiol-based antioxidant, upregulates GSH/GSSG and reduces the respiratory burst and MAPK- and NF-kB-mediated pro-inflammatory cytokine release during inflammation produced by muscle injury [30, 63]. Information derived from this study will elucidate the redox-dependent regulation of intracellular signaling pathways involved in SCs regulation and muscle healing in human skeletal muscle. Therefore, the results of the proposed study should provide information about possible nutritional and/or pharmaceutical interventions to promote SCs function and increase skeletal muscle’s healing potential.

### Trial status

The trial has not yet commenced. Participant recruitment is ongoing.

## Additional file

Additional file 1: Standard Protocol Items: Recommendations for Interventional Trials (SPIRIT) 2013 Checklist: recommended items to address in a clinical trial protocol and related documents. (DOCX 50 kb)

## Data Availability

All primary and secondary outcome data will be published in data depositories. The study adheres to the SPIRIT 2013 Checklist (Additional file 1).

## Abbreviations

BMI: Body mass index
CAT: Catalase
CD11b: Cluster of differentiation 11b marker
CD206: Cluster of differentiation 206 marker
CK: Creatine kinase
CRP: C-reactive protein
CSA: Cross-sectional area
DAPI: 4’,6-diamidino-2-phenylindole
DOMS: Delayed onset of muscle soreness
DXA: Dual energy X-ray absorptiometry
EDTA: Ethylenediaminetetraacetic acid
EIMD: Exercise-induced muscle damage
GAPDH: Glyceraldehyde 3-phosphate dehydrogenase
GPX-3: Glutathione peroxidase 3
GSH: Reduced glutathione
GSSG: Oxidized glutathione
H&E: Hematoxylin and eosin
Hb: Hemoglobin
HR: High respondents
IGF-1: Insulin-like growth factor 1
IL-10: Interleukin 10
IL-6: Interleukin 6
IL-8: Interleukin 8
KE: Knee extensors
LR: Low respondents
M1: Pro-inflammatory-phenotype macrophages
M2: Anti-inflammatory-phenotype macrophages
MAPK: Mitogen-activated protein kinase
MDA: Malondialdehyde
MRFs: Myogenic regulatory factors
mRNA: Messenger RNA
Myf4: Myogenic factor 4
Myf5: Myogenic factor 5
MyoD: Myogenic determination factor
NAC: *N*-acetylcysteine
NF-kB: Nuclear factor kappa-light-chain-enhancer of activated B cells
OCT: Optimal cutting temperature
OXS: Oxidative stress
PA: Physical activity
Pax7: Paired box protein
PBS: Phosphate buffer saline
PC: Protein carbonyls
RBCs: Red blood cells
RCT: Randomized clinical trial
RDA: Recommended dietary allowance
RMR: Resting metabolic rate
ROS: Reactive oxygen species
RT-PCR: Real-time polymerase chain reaction
SCs: Satellite cells
SOD-2: Superoxide dismutase 2
TAC: Total antioxidant capacity
TBS: Tris-buffer saline
TNF-α: Tumor necrosis factor alpha
Trx1: Thioredoxin 1
VO_2max_: Maximal oxygen consumption
